# A True Aneurysm of the Zygomatic Orbital Artery: First Case Report in the Literature

**DOI:** 10.1155/2012/873168

**Published:** 2012-02-20

**Authors:** Valentina Molinaro, Elisa Pagliasso, Gianfranco Varetto, Claudio Castagno, Lorenzo Gibello, Pietro Rispoli

**Affiliations:** School of Vascular Surgery, Molinette Hospital, University of Turin, Corso Bramante 88/90, 10126, Turin, Italy

## Abstract

A 33-year-old man presented with a pulsatile mass in the left temporal region; about 1 year before the current presentation, the swelling had arisen on the upper lateral border of the orbital arch and increased in recent months. His medical history was negative for accidental or iatrogenic head injury. Color echo Doppler and angio-computed tomography demonstrated a fusiform aneurysm of the zygomatic orbital artery, a branch of the superficial temporal artery. Blood tests were negative for human immunodeficiency virus (HIV), hepatitis C (HCV), and hepatitis B (HBV) markers. Aneurysmectomy under local anesthesia was performed. Histology of the surgical specimen confirmed the diagnosis of a true aneurysm measuring 8.4 × 5.7 mm, which showed no atherosclerotic degeneration of the vessel walls; the lumen was filled by a recent thrombus but without inflammatory cells. Surgical treatment is indicated for the prevention of rupture, the relief of pain when present, and the removal of facial defects. To the authors' knowledge, this is the first case in the literature of a true aneurysm of the zygomatic orbital artery.

## 1. Introduction

The superficial temporal artery (STA) is one of the terminal branches of the external carotid artery. At the zygomatic process, it divides into a frontal (anteriorly) and a parietal (posteriorly) branch. Just before its bifurcation, the transversal facial artery, the anterior auricular arteries, and the zygomatic orbital artery branch out of it. The zygomatic orbital artery runs parallel to the zygomatic arch and anastomizes with the palpebral artery and the lacrimal artery, both branches of the ophthalmic artery. In 22% of cases, the zygomatic orbital artery is absent [1].

Aneurysms of the STA account for nearly all posttraumatic pseudoaneurysms (accidental, postsurgical, following hair transplantation, etc.) among the over 400 cases described in the literature [2]. In contrast, true spontaneous aneurysms are exceptionally rare and their etiology remains unclear. STA aneurysms from a variety of causes have been described: atherosclerosis [3], human immunodeficiency virus (HIV) infection [4], idiopathic, or associated with cerebral aneurysms [5]. For these reasons and owing to its anatomic contiguity with the STA, a true aneurysm of the zygomatic orbital artery, perhaps the first to be reported here, may be compared to an STA aneurysm.

## 2. Case Report

**Figure 1:**
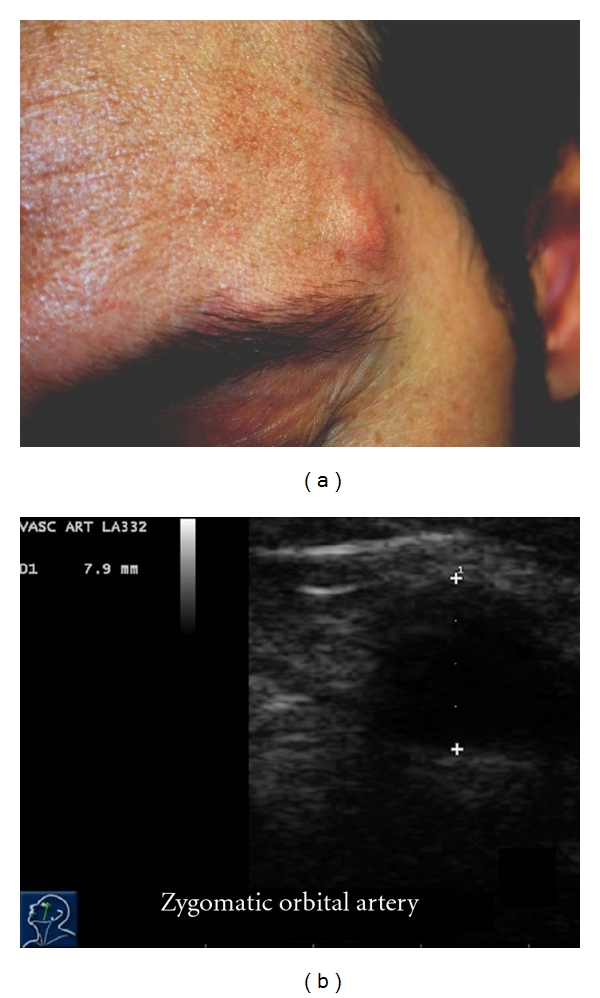
(a) Pulsatile subcutaneous mass on the upper lateral border of the left orbital arch. (b) Cross section of zygomatic orbital artery aneurysm at preoperative echo color Doppler.

**Figure 2:**
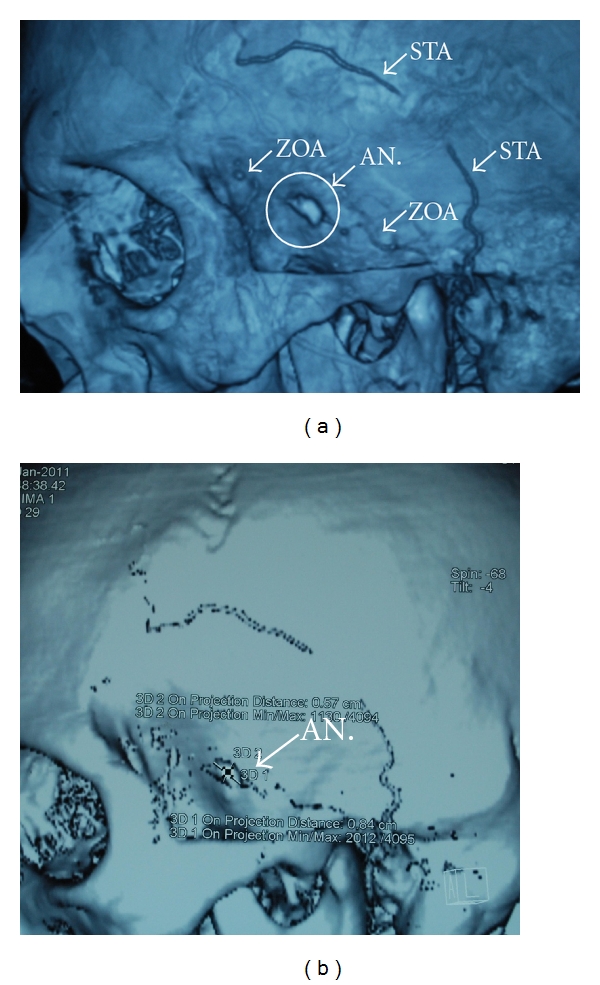
(a) 3D CT scan reconstruction showing the aneurysm of the zygomatic orbital artery, a branch of the left superficial temporal artery (arrow). STA denotes superficial temporal artery; ZOA zygomatic orbital artery, (b) Angio-CT scan of the aneurysm (approximately 8.45 × 5.7 mm).

**Figure 3:**
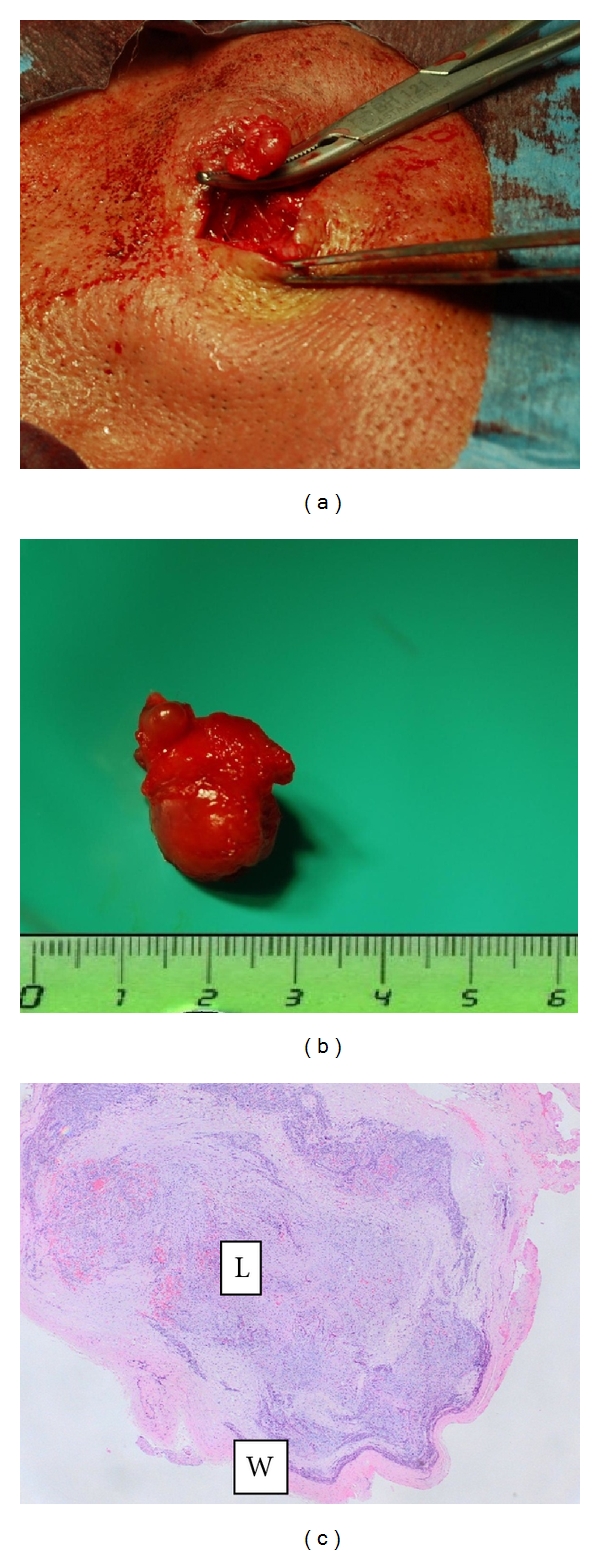
(a) Excision of the aneurysm, (b) resected aneurysm, (c) microphotograph of the resected mass. L: lumen filled with fresh, organized thrombus; W: wall of the aneurysm consisting of the intima, media, and adventitia.

A 33-year-old man, in otherwise good health, was seen because of a subacute enlargement of a pulsatile mass in the left temporal region. His medical history was negative for accidental and iatrogenic head injury, headache, and cardiovascular risk factors. Physical examination revealed a small pulsatile mass on the upper lateral border of the left orbital arch (Figure 1(a)); the swelling was nontender and elastic and the overlying skin was slightly inflamed. Echo color Doppler demonstrated triphasic flow at the lesion site, which was hypoechogenic compared to the surrounding region; the true lumen was only partially occluded by an apparently recent thrombus (Figure 1(b)). An atypical presentation of Horton's arteritis was initially considered preoperatively, but was ruled out due to the absence of inflammatory cells at histologic examination. 3D angio-computed tomography (CT) of the intracranial and extracranial vessels and the aortic arch showed an aneurysm of a branch of the left STA, anatomically identified as the zygomatic orbital artery, measuring about 8.4 × 5.7 mm (Figures 2(a) and 2(b)). Neither cerebral aneurysms nor lesions of the aortic arch were detected. Blood inflammatory marker testing revealed no elements to support the hypothesis of systemic arteritis or autoimmune disease. Aneurysmectomy under local anesthesia was performed (Figures 3(a) and 3(b)) through a small incision at the upper margin of the zygomatic arch, paying great attention in avoiding to injure the auriculotemporal nerve which passes nearby. The postoperative course was uneventful; no headache, loco-regional ischemia, or neurologic lesions developed; the patient was discharged on postoperative day 1. Histology and immuno-histochemical analysis of the surgical specimen confirmed the diagnosis of a fusiform aneurysm (Figure 3(c)); there was no atherosclerotic degeneration; the lumen was found to contain a recent thrombus but without inflammatory cells; an histologic pattern typical of Horton's arteritis or of other types of inflammatory arteritis of was ruled out. Testing for bacteria, fungal hypha, type IV collagen, and Pan-Keratin was negative.

## 3. Discussion

To date, no cases have been reported of spontaneous aneurysm of the zygomatic orbital artery, one of the branches of the STA. True aneurysms of the STA are fairly rare, whereas those from injury are much more common (about 90% of cases) [6] because the STA passes over bone and is covered by a thin layer of cutaneous and subcutaneous tissues. Posttraumatic dilatations of the facial vasculature are often pseudoaneurysms and generally appear within 2 months following the event of injury [7].

Spontaneous or nontraumatic aneurysms may be caused by atherosclerosis [3], congenital alterations in the internal elastic lamina [8], or chronic arteritis [9]. Cases have been reported of extracranial aneurysms associated with HIV infection [4, 7], although it is unclear whether aneurysmatic degeneration is due to the virus or to a bacterial superinfection associated with immunodeficiency.

The differential diagnosis includes hematoma, cysts, reactive lymph node, lipoma, and arteriovenous fistula. On palpation, the aneurysm can be appreciated as a pulsatile mass; pulsation of the swelling will diminish or disappear when the artery is compressed proximal to the mass. The presumptive diagnosis can be confirmed by color echo Doppler, which shows triphasic blood flow within the lesion. Second level diagnostic imaging studies (CT scan and magnetic resonance angiography) are useful for confirming the diagnosis and to detect possibly associated intra- and extracranial aneurysms [2].

Scrupulous history taking ruled out previous head injury (accidental, sports-related or iatrogenic) in our patient. Histology demonstrated that the aneurysm involved all three vessel wall layers (Figure 3(c)), without signs of atherosclerotic degeneration or chronic inflammation. Blood tests were negative for HIV, HCV, and HBV markers. The possible cause of the aneurysm remains unknown.

Owing to its low incidence, the natural history of true aneurysms of the STA is unclear, whereas pseudoaneurysms are known to tend to progress to acute thrombosis or rupture [7]. The literature does not indicate a threshold lesion size above which surgery can be recommended [10]. To date, the indications for the treatment of true aneurysms of the STA and its branches include pain due to nerve compression, enlargement of the mass, and self-perceived facial imbalance. Altered facial appearance was, in fact, the principal reason our patient sought medical attention. The treatment options described in the literature include endovascular techniques and conventional aneurysmectomy. Embolization with thrombin [11] or microcoils [12] is not indicated if the vessel lumen is filled by thrombotic material, as in our patient. Furthermore, endovascular treatment carries the risk of incomplete vessel occlusion or recanalization, resulting in procedure failure [11]. This is further burdened by a lack of or an extremely slow reduction of the swelling, the underlying cause of unacceptable cosmesis [11]. For these reasons, we elected to perform aneurysmectomy under local anesthesia. The procedure was completed within a few minutes and was well tolerated. The intradermal suture with absorbable suture produced an excellent cosmetic result. The patient will be clinically followed up once a year for the next three years.

## 4. Conclusions

True spontaneous STA aneurysms are a rare occurrence and are nearly always posttraumatic pseudoaneurysms. Careful history taking and clinicodiagnostic assessment are key to establishing a correct differential diagnosis.

Therapeutic treatment is indicated for preventing rupture, relieving pain, and restoring cosmesis in particular. Surgery is the first choice of treatment for STA aneurysms, given the low risk and high success rates, whereas selective embolization may offer an alternative surgical option in some cases. In our opinion, however, especially in cases such as ours, the zygomatic orbital artery is not amenable to endovascular treatment because of its extremely small caliber (±1 mm) and because of the high risk of failure to remove the facial defect caused by the aneurysm.

This case holds interest because, to the authors' knowledge, it is the first in the literature of a spontaneous aneurysm of the zygomatic orbital artery without traumatic origin.
